# A New Pipeline for the Normalization and Pooling of Metabolomics Data

**DOI:** 10.3390/metabo11090631

**Published:** 2021-09-17

**Authors:** Vivian Viallon, Mathilde His, Sabina Rinaldi, Marie Breeur, Audrey Gicquiau, Bertrand Hemon, Kim Overvad, Anne Tjønneland, Agnetha Linn Rostgaard-Hansen, Joseph A. Rothwell, Lucie Lecuyer, Gianluca Severi, Rudolf Kaaks, Theron Johnson, Matthias B. Schulze, Domenico Palli, Claudia Agnoli, Salvatore Panico, Rosario Tumino, Fulvio Ricceri, W. M. Monique Verschuren, Peter Engelfriet, Charlotte Onland-Moret, Roel Vermeulen, Therese Haugdahl Nøst, Ilona Urbarova, Raul Zamora-Ros, Miguel Rodriguez-Barranco, Pilar Amiano, José Maria Huerta, Eva Ardanaz, Olle Melander, Filip Ottoson, Linda Vidman, Matilda Rentoft, Julie A. Schmidt, Ruth C. Travis, Elisabete Weiderpass, Mattias Johansson, Laure Dossus, Mazda Jenab, Marc J. Gunter, Justo Lorenzo Bermejo, Dominique Scherer, Reza M. Salek, Pekka Keski-Rahkonen, Pietro Ferrari

**Affiliations:** 1 Nutrition and Metabolism Branch, International Agency for Research on Cancer (IARC-WHO), 69008 Lyon, France; hism@fellows.iarc.fr (M.H.); rinaldis@iarc.fr (S.R.); breeurm@students.iarc.fr (M.B.); GicquiauA@iarc.fr (A.G.); hemonb@iarc.fr (B.H.); dossusl@iarc.fr (L.D.); jenabm@iarc.fr (M.J.); gunterm@iarc.fr (M.J.G.); SalekR@iarc.fr (R.M.S.); KeskiP@iarc.fr (P.K.-R.); ferrarip@iarc.fr (P.F.); 2 Department of Public Health, Aarhus University Bartholins Alle 2, DK-8000 Aarhus, Denmark; ko@ph.au.dk; 3 Danish Cancer Society Research Center, DK-2100 Copenhagen, Denmark; annet@CANCER.DK (A.T.); agnrha@cancer.dk (A.L.R.-H.); 4 UVSQ, Inserm, CESP U1018, “Exposome and Heredity” Team, Université Paris-Saclay, Gustave Roussy, 94800 Villejuif, France; Joseph.ROTHWELL@gustaveroussy.fr (J.A.R.); lucie.lecuyer@inserm.fr (L.L.); gianluca.severi@inserm.fr (G.S.); 5 Department of Statistics, Computer Science, Applications “G. Parenti”, University of Florence, 50134 Florence, Italy; 6 Division of Cancer Epidemiology, German Cancer Research Center (DKFZ), 69120 Heidelberg, Germany; r.kaaks@dkfz-heidelberg.de (R.K.); t.johnson@Dkfz-Heidelberg.de (T.J.); 7 Department of Molecular Epidemiology, German Institute of Human Nutrition Potsdam Rehbruecke, Arthur-Scheunert-Allee 114-116, 14558 Nuthetal, Germany; mschulze@dife.de; 8 Institute of Nutritional Science, University of Potsdam, Arthur-Scheunert-Allee 114-116, 14558 Nuthetal, Germany; 9 Cancer Risk Factors and Life-Style Epidemiology Unit, Institute for Cancer Research, Prevention and Clinical Network (ISPRO), 50139 Florence, Italy; d.palli@ispro.toscana.it; 10 Epidemiology and Prevention Unit Department of Research, Fondazione IRCCS—Istituto Nazionale dei Tumori, 20133 Milan, Italy; Claudia.Agnoli@istitutotumori.mi.it; 11 Dipartimento di Medicina Clinica e Chirurgia, Federico II University, 80131 Naples, Italy; salvatorepanico2@gmail.com; 12 Cancer Registry and Histopathology Department, Provincial Health Authority (ASP 7), 97100 Ragusa, Italy; rtuminomail@gmail.com; 13 Department of Clinical and Biological Sciences, University of Turin, 10043 Orbassano, Italy; fulvio.ricceri@unito.it; 14 Unit of Epidemiology, Regional Health Service ASL TO3, 10095 Grugliasco, Italy; 15 National Institute for Public Health and the Environment, Centre for Nutrition, Prevention and Health Services, Antonie van Leeuwenhoeklaan 9, 3721 MA Bilthoven, The Netherlands; monique.verschuren@rivm.nl (W.M.M.V.); peter.engelfriet@rivm.nl (P.E.); 16 Julius Center for Health Sciences and Primary Care, University Medical Center Utrecht, 3584 CG Utrecht, The Netherlands; n.c.onland@umcutrecht.nl (C.O.-M.); R.C.H.Vermeulen@uu.nl (R.V.); 17 Institute for Risk Assessment Sciences, Division of Environmental Epidemiology, Utrecht University, 3584 CM Utrecht, The Netherlands; 18 Department of Community Medicine, Faculty of Health Sciences, UiT The Arctic University of Norway, P.O. Box 6050, 9037 Tromsø, Norway; therese.h.nost@uit.no (T.H.N.); ilona.urbarova@uit.no (I.U.); 19 Unit of Nutrition and Cancer, Cancer Epidemiology Research Programme, Catalan Institute of Oncology, Bellvitge Biomedical Research Institute (IDIBELL), 08908 L’Hospitalet de Llobregat, Spain; raulzamoraros@gmail.com; 20 Escuela Andaluza de Salud Pública (EASP), 18011 Granada, Spain; miguel.rodriguez.barranco.easp@juntadeandalucia.es; 21 Instituto de Investigación Biosanitaria ibs.GRANADA, 18012 Granada, Spain; 22 Centro de Investigación Biomédica en Red de Epidemiología y Salud Pública (CIBERESP), 28029 Madrid, Spain; epicss-san@euskadi.eus (P.A.); jmhuerta.carm@gmail.com (J.M.H.); me.ardanaz.aicua@navarra.es (E.A.); 23 Ministry of Health of the Basque Government, Sub-Directorate for Public Health and Addictions of Gipuzkoa, 20013 San Sebastián, Spain; 24 Biodonostia Health Research Institute, Group of Epidemiology of Chronic and Communicable Diseases, 20014 San Sebastián, Spain; 25 Department of Epidemiology, Murcia Regional Health Council, IMIB-Arrixaca, 30007 Murcia, Spain; 26 Navarra Public Health Institute, 31003 Pamplona, Spain; 27 IdiSNA, Navarra Institute for Health Research, 31008 Pamplona, Spain; 28 Department of Clincal Sciences, Lund University, SE-21 428 Malmö, Sweden; olle.melander@med.lu.se; 29 Department of Emergency and Internal Medicine, Skåne University Hospital, SE-20 502 Malmö, Sweden; 30 Department of Immunotechnology, Lund University, SE-22 100 Lund, Sweden; filip.ottosson@med.lu.se; 31 Department of Radiation Sciences, Oncology, Umeå University, SE-901 87 Umeå, Sweden; linda.vidman@umu.se (L.V.); matilda.rentoft@umu.se (M.R.); 32 Cancer Epidemiology Unit, Nuffield Department of Population Health, University of Oxford, Oxford OX3 7LF, UK; js@clin.au.dk (J.A.S.); ruth.travis@ndph.ox.ac.uk (R.C.T.); 33 International Agency for Research on Cancer, World Health Organization, 69008 Lyon, France; WeiderpassE@iarc.fr; 34 Genomic Epidemiology Branch, International Agency for Research on Cancer (IARC-WHO), 69008 Lyon, France; JohanssonM@iarc.fr; 35 Statistical Genetics Group, Institute of Medical Biometry, University of Heidelberg, 69120 Heidelberg, Germany; lorenzo@imbi.uni-heidelberg.de (J.L.B.); scherer@imbi.uni-heidelberg.de (D.S.)

**Keywords:** cancer epidemiology, normalization, pooling, technical variability, metabolomics, metabolites

## Abstract

Pooling metabolomics data across studies is often desirable to increase the statistical power of the analysis. However, this can raise methodological challenges as several preanalytical and analytical factors could introduce differences in measured concentrations and variability between datasets. Specifically, different studies may use variable sample types (e.g., serum versus plasma) collected, treated, and stored according to different protocols, and assayed in different laboratories using different instruments. To address these issues, a new pipeline was developed to normalize and pool metabolomics data through a set of sequential steps: (i) exclusions of the least informative observations and metabolites and removal of outliers; imputation of missing data; (ii) identification of the main sources of variability through principal component partial R-square (PC-PR2) analysis; (iii) application of linear mixed models to remove unwanted variability, including samples’ originating study and batch, and preserve biological variations while accounting for potential differences in the residual variances across studies. This pipeline was applied to targeted metabolomics data acquired using Biocrates AbsoluteIDQ kits in eight case-control studies nested within the European Prospective Investigation into Cancer and Nutrition (EPIC) cohort. Comprehensive examination of metabolomics measurements indicated that the pipeline improved the comparability of data across the studies. Our pipeline can be adapted to normalize other molecular data, including biomarkers as well as proteomics data, and could be used for pooling molecular datasets, for example in international consortia, to limit biases introduced by inter-study variability. This versatility of the pipeline makes our work of potential interest to molecular epidemiologists.

## 1. Introduction

Metabolomics is a powerful tool for investigating candidate etiological pathways for chronic diseases [1,2,3,4]. Using either untargeted or targeted (via sets of pre-defined annotated metabolites) approaches, prior metabolomics studies have identified metabolites associated with the risk of several chronic conditions, including type-2 diabetes (T2D) [5], cardiovascular diseases (CVD) [6], and cancer [7,8,9]. Metabolomics has also been used to characterize specific signatures of anthropometric measures and lifestyle exposures, including body mass index (BMI) [7,10], adherence to a Mediterranean diet [6], and coffee consumption [5], as a way to investigate candidate biological mechanisms underpinning the relationship between these exposures and chronic diseases.

As with other-omics technologies, pre-processing metabolomics data is critical before relating them to phenotypes, such as cancer endpoints or lifestyle exposures [11,12]. After a matrix of *p* metabolites (or features) measured in *n* samples has been generated, pre-processing usually involves (i) feature and sample filtering, where low-quality features and samples are excluded, (ii) data imputation, to take care of missing values, and (iii) data normalization, to correct for sources of unwanted variation in metabolomics data, such as batch effects and other factors related to the handling of samples [11,13,14,15,16]. Following the success of data acquisition efforts in large-scale epidemiological investigation, collaborative consortia have been put in place, offering the possibility to pool metabolomics data acquired in different studies in order to increase sample size and range of biological variation, and eventually enhance the statistical power of the analysis. However, pooling metabolomics data across studies raises methodological challenges as several preanalytical and analytical factors can induce differences in metabolite measurements and unwanted variability between datasets. Specifically, sample types (e.g., serum versus plasma), fasting status of the participant, and any other elements related to sampling conditions, sample treatment, and storage represent preanalytical factors, while analytical factors include information on the organization of samples in batches, the acquisition instrument, the acquisition time (i.e., time at which the sample was assayed), and the laboratory [17]. Correcting for these sources of variations is crucial in order to conduct accurate statistical analyses on pooled datasets.

Data on common quality controls assayed in all studies and/or reference assay data from a subset of samples in each study can be used for normalization [17,18]. However, these data are not always available in large international investigations and consortia. Accordingly, we developed a pipeline for the normalization and pooling of metabolomics data acquired in different studies that does not require data on quality controls or reference assay data, which covers four main steps. First, data cleaning identified and removed features and samples exceeding certain thresholds of missingness and outlying samples [11,16]. Second, the remaining missing values were imputed within each study using information on limits of detection and quantification when available and appropriate, and measurements were log-transformed to reduce skewness. Third, the principal component partial R-square (PC-PR2) technique was implemented to identify sources of variation in the metabolomics data [13]. Last, mixed effect models were used to correct for unwanted variability while preserving biological variability [14]. The ComBat method [19] implemented in the R sva package [20] and a PCA-based method [21,22] were also implemented for comparison. Our pipeline was applied to targeted metabolomics data acquired in eight case-control studies nested within the European Prospective Investigation into Cancer and Nutrition (EPIC) [23]. Comprehensive analytical and graphical examinations of measurements were performed to assess whether different normalization approaches improved the comparability of metabolomics data. For illustration, metabolomics data were pooled and related to study participants’ BMI. 

## 2. Results

### 2.1. Description of the Study Population 

Targeted metabolomics data acquired within the EPIC study and centralized at the International Agency for Research on Cancer (IARC) included 16,060 pre-diagnostic blood samples originating from eight case-control studies nested within EPIC (details in Section 4.1) on seven types of cancer: breast cancer (one study denoted by BREA; *n* = 3172 samples) [8], endometrial cancer (ENDO; *n* = 1706) [24], gallbladder cancer (GLBD; *n* = 112), liver cancer (LIVE; *n* = 596) [25], kidney cancer (KIDN; *n* = 1213) [26], prostate cancer (PROS; *n* = 6020) [9,27], and colorectal cancer (two studies denoted by CLRT1 and CLRT2; *n* = 946 and *n* = 2295, respectively). As displayed in Table 1, samples collected at recruitment were assayed at IARC for BREA, LIVE, KIDN, PROS, and CLRT1, at the Helmholtz Zentrum (München, Germany) for CLRT2 and GLBD, and at the Imperial College London (UK) for ENDO. Across all studies, measurements of a total of 171 metabolites were acquired using either the AbsoluteIDQ p180 or the AbsoluteIDQ p150 (for CLRT2 only) commercial kit (Biocrates Life Science AG, Innsbruck Austria), following the procedure recommended by the vendor. As displayed in Table 1, samples were assayed on different liquid chromatography (LC) and mass spectrometry (MS) instruments across the different studies, but each study used one single pair of LC-MS instruments for all samples. Samples were mostly either serum or citrate plasma, and samples within one study all originated from the same type of blood matrix, except in BREA and GLBD where samples from Swedish participants originated from a different blood matrix compared to the other participants. For these two studies, samples assayed within each batch all originated from the same blood matrix (not shown). Samples were assayed between 2013 and 2018. The pipeline detailed in Section 4.2 was applied to the (*n* × p) matrix with *n* = 16,060 samples and the *p* = 118 metabolites measured in all studies. Specifically, they included 13 metabolites (amino acids) measured by a quantitative LC-MS/MS method (liquid chromatography coupled with tandem mass spectrometry) and 105 metabolites (76 glycerophospholipids, 12 sphingolipids, 16 acylcarnitines, and 1 hexose, the sum of six-carbon sugars) acquired by a semi-quantitative FIA-MS/MS method (flow injection analysis coupled with tandem mass spectrometry, one-point calibration, no individual internal standards). 

### 2.2. Data Cleaning and Imputation

For the exclusion of metabolites and samples exceeding a given threshold of missingness, we applied the method described in Section 4.2.1 with a threshold set to 20% and with missing values defined as “fully missing” values only, i.e., not including values out of measurable range. Among the 118 metabolites originally retained for the analysis, the acylcarnitine C4-OH (C3-DC) was the only one with a fully missing value in more than 20% of the samples of at least one study (PROS), and was excluded. Among the 16,060 samples originally retained for the analysis, none was excluded because of exceeding 20% of fully missing values, eight were excluded because they were measured in batches with less than 10 samples, and two were excluded because they were considered outliers after a principal component analysis (PCA). Thus, the final study population included 16,050 samples, for which measurements of 117 metabolites were included (Supplementary Table S1). Out of the 1,877,850 corresponding measurements, 1066 were fully missing and 63,564 were out of the measurable range: specifically, 63,044 were below a known LOD (limit of detection, applicable to acylcarnitines, glycerophospholipids, hexose, and sphingolipids), 517 below a known LLOQ (lower limit of quantification, applicable to amino acids), 2 above a known ULOQ (upper limit of quantification), and 1 below an unknown LOD. All these 1066 + 63,564 = 64,630 missing values were imputed as described in Section 4.2.2, and concentration values were log-transformed. 

### 2.3. Identification of Major Sources of Variations 

**Figure 1:**
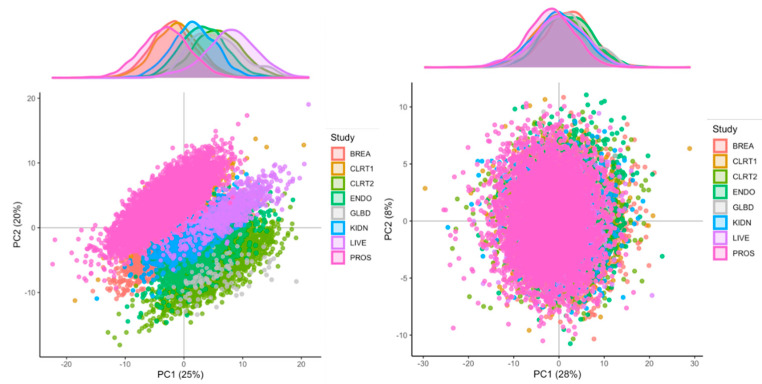
Results from the principal component analysis (PCA); **left panel**: PCA of the imputed data (i.e., before the normalization step); **right panel**: PCA of the normalized data. In each plot, the *x*−axis and *y*−axis represent the projections of the metabolomics measurements on the first (PC1) and on the second (PC2) principal components, respectively. Proportions of the total variation explained by each component are given in parenthesis in the axis labels.

**Figure 2:**
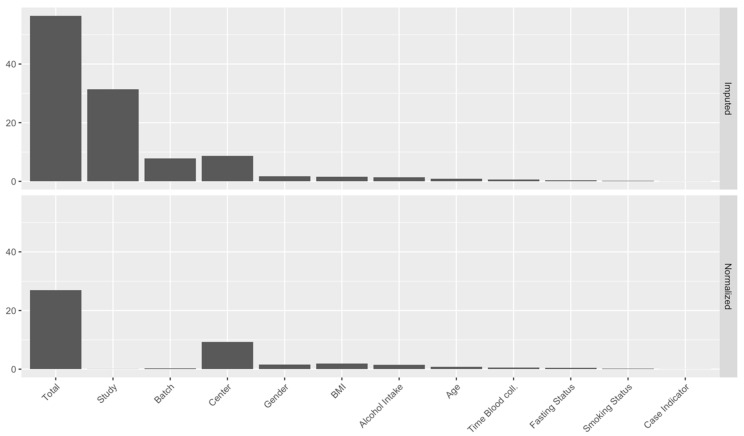
Results from the PC-PR2 analysis of the imputed data (i.e., before the normalization step; (**top**)) and the normalized data (**bottom**). In each plot, the *y*−axis represents the proportion (expressed as a percentage) of the total variation of the metabolomics measurements explained by each variable represented on the *x*−axis, together with their total.

As displayed in Figure 1 (left panel), the projection of the measurements on the first two principal components of the PCA were strongly clustered by study, suggesting the presence of systematic sources of heterogeneity across studies. The PC-PR2 method was applied to assess the proportion of the overall variation in the metabolomics data that was explained by a predefined list of variables, including (i) participants’ characteristics, i.e., study center, gender, case-control indicator, age, BMI, alcohol intake, smoking status, and (ii) three variables describing possible preanalytical and analytical sources of unwanted variations: fasting status, time of the day of blood collection, study, and batch, with batch nested within study. As shown in Figure 2 (top panel), the PC-PR2 analysis indicated that these variables together explained more than 55% of the total variation of the metabolomics measurements before normalization. Study explained 31% of the total variation, while batch within study explained about 8%. Study center explained about 9% of the total variation, and gender, BMI, and alcohol intake explained about 2%, 2%, and 1%, respectively. Fasting status, time of blood collection, age at recruitment, smoking status, and case-control status all explained less than 1% of the total variation. 

### 2.4. Normalization of the Measurements

**Figure 3:**
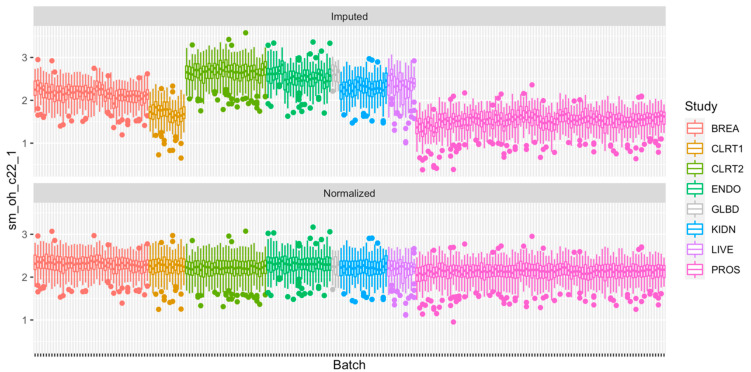
Boxplots of SM OH C22:1 within each of the batches of the eight case-control studies for the imputed data (**top**) and the normalized data (**bottom**). Dots indicate measurements out of the interval (q1 − 1.5 × IQR, q3 + 1.5 × IQR) with q1 and q3 being the first and third quartile, respectively, and IQR = q3 − q1 the interquartile range. In each plot, the *y*−axis represents the value of the measurement and the *y*−axis represents each batch, arranged by study.

Based on PC-PR2 analysis, metabolite concentrations were normalized using the method described in Section 4.2.4 to correct for variation due to study and batch, and preserve the variation due to study center, gender, BMI and alcohol intake. These latter four variables were all unequally distributed across studies and batches (not shown). They were included as fixed effects in matrix Z (Equation (2) in Section 4.2.4; otherwise, some of the variation they explain would be removed because of the adjustment for study and batch, while study and batch within study were modeled as random effects in matrix X. Other variables studied in the PC-PR2 analysis were not included in matrix X or Z as they contributed very little to the total variation. Heteroscedastic metabolite-specific mixed models with a study-specific variance component were used, although homoscedastic models produced very similar results (not shown). The PCA of normalized data (Figure 1; right panel) indicated that the projections on the first two principal components were not clustered by study anymore, and measurements’ distribution largely overlapped. Data from PROS (men only) were slightly shifted to the left and data from BREA and ENDO (women only) were shifted to the right, suggesting that the normalization preserved some variation due to gender overall. For illustration, the distribution of one semi-quantified metabolite, SM OH C22:1, was computed within batches and across studies, for the imputed and the normalized measurements (Figure 3). Imputed data displayed a study effect, with concentration levels of SM OH C22:1 in the CLRT2, ENDO, GLBD, KIDN, and LIVE studies substantially larger than those in BREA, CLRT1, and PROS. A remarkable batch effect was observed within some studies, e.g., BREA. After normalization, the distributions were very similar across batches and studies. Again, the distribution was slighty shifted downward for concentration levels in PROS (men only), and upward in BREA and ENDO (women only), compared to the other five studies CLRT1, CLRT2, GLBD, KIDN, and LIVE (which included both men and women), confirming that the normalization preserved some variation due to gender for this particular metabolite. The PC-PR2 analysis of normalized data (Figure 2, bottom panel) confirmed that normalization removed unwanted sources of variation (batch and study), but kept most variability attributed to participants’ characteristics. Complementary PC-PR2 analysis showed that blood matrix and LC-MS instruments contributed to less than 0.1% of the total variation after normalization (results not shown). Compared to our approach, the PCA-based method produced rather different results for many metabolites, while ComBat [19] produced very similar results for all metabolites with the exception of most acylcarnitines and the glycerophospholipid PC aa C40:1 (Supplementary Figures S1 and S2). 

### 2.5. Technical Reproducibility of Measurements before and after Normalization

**Figure 4:**
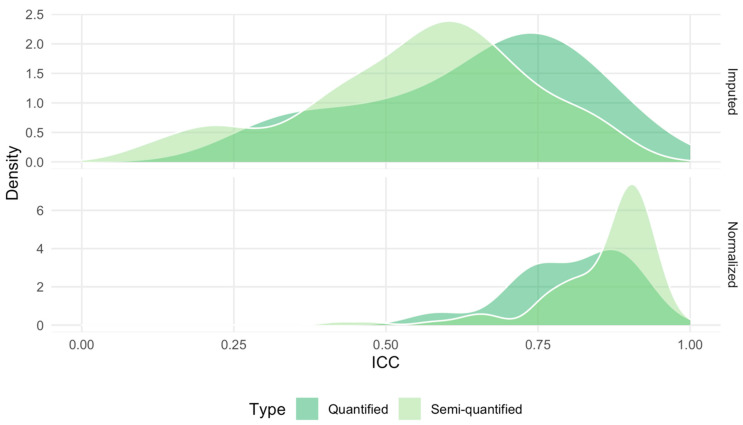
Distribution of the intraclass correlation coefficient (ICC) for quantified and semi-quantified metabolites, before (**top**) and after (**bottom**) normalization. In each plot, the *x*–axis represents the value of the ICC and the *y*–axis the value of the estimated density.

**Figure 5:**
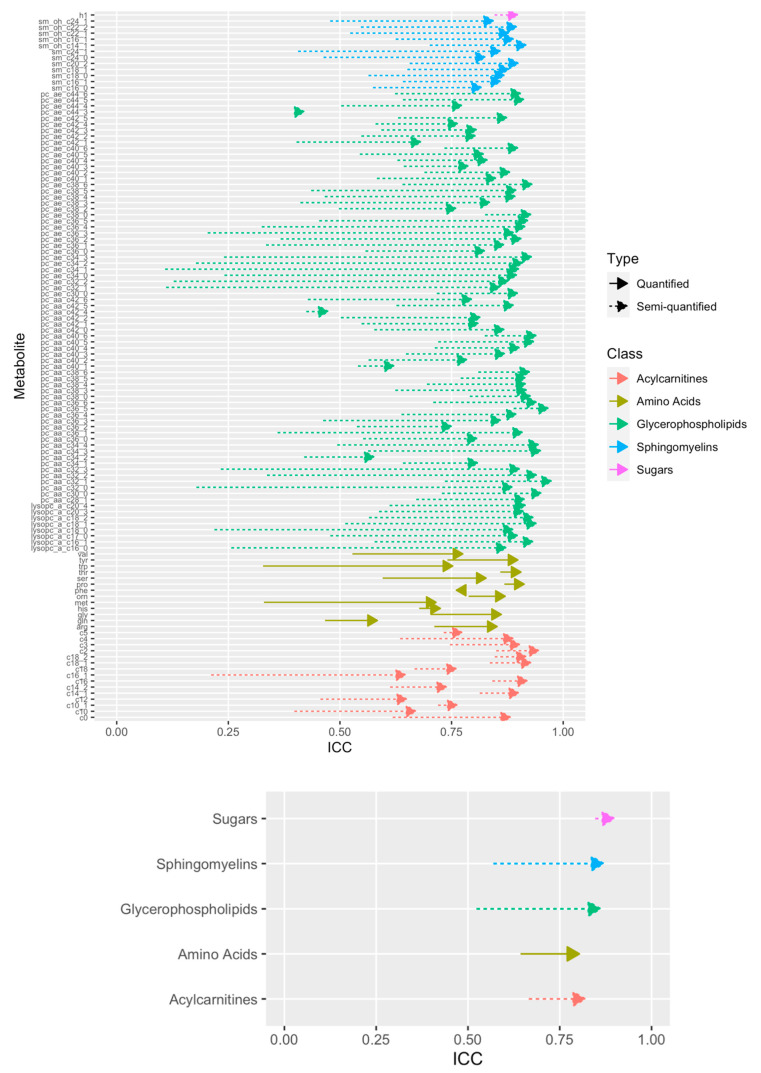
Metabolite-specific ICC values before and after normalization (**top**) and average ICC values for each class of metabolites before and after normalization (**bottom**). For each arrow, its origin represents the ICC value before normalization, and its peak represents the ICC value after normalization. In each plot, the *x*−axis represents the ICC value, and the *y*−axis each particular metabolite (**top**) or class of metabolites (**bottom**).

Intraclass correlation coefficients (ICC) were computed for each metabolite to assess their technical reproducibility, using measurements from 2 × 219 = 438 duplicate samples, i.e., samples measured once in two different studies (2 × 147 samples; see Supplementary Table S2) or in two different batches of the prostate study (2 × 72 samples), as detailed in Section 4.3. Figure 4 shows the distributions of ICCs for the semi-quantified (lipids, acylcarnitines, and hexose) and quantified (amino acids) metabolites, before and after normalization. Normalization shifted the distribution of ICCs upward for semi-quantified metabolites. The distribution of quantified metabolites did not shift as much, but the variability narrowed down, with no ICC value lower than 0.50. Figure 5 shows the effect of the normalization on the ICC of each individual metabolite (top), and on the average ICC for each class of metabolites (bottom). Before normalization, 101 (86%) metabolites (92 semi-quantified, 9 quantified) had ICC values lower than 0.75, among which 38 (32%: 35 semi-quantified, 3 quantified) had ICC values lower than 0.5. After normalization, only twelve metabolites (10%: 9 semi-quantified, 3 quantified) had ICC values lower than 0.75, among which only two semi-quantified metabolites had ICC lower than 0.50. Moreover, class-specific averaged ICC values were consistently improved by the normalization, in particular for glycerophospholipids and sphyngomyelins. Similar results were observed when normalization was performed with ComBat [19], yet ICCs were lower than those obtained when using our approach, especially for acylcarnitines (Supplementary Figure S3). ICCs were even lower when normalization was performed with the PCA-based method (Supplementary Figure S4). The same analysis was restricted to the 2 × 57 = 114 duplicate samples acquired in two studies from serum and citrate plasma. As displayed in Supplementary Figure S5, ICC values were lower than 0.5 for 69 metabolites (59%) and 4 metabolites (3%), before and after normalization, respectively, with ICC values greater than 0.75 for 91 metabolites (78%) after normalization. 

### 2.6. Impact of Normalization When Relating a Given Phenotype to the Metabolites 

**Figure 6:**
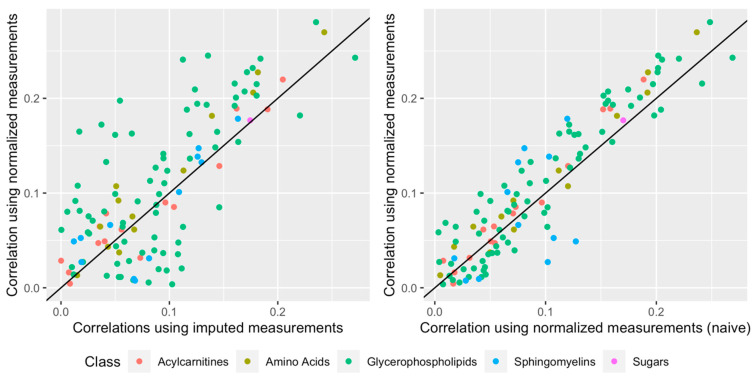
Correlations (absolute values) between BMI and the 117 metabolites in control samples. The *y*−axis represents values computed with normalized measurements produced by our approach, while the *x*−axis represents values computed with imputed (non-normalized) measurements (**left**), and normalized measurements produced by the simpler normalization approach (**right**), which corrected for study and batch effects without specifically attempting to preserve variation due to study center, BMI, gender, and alcohol intake.

The relationship between the metabolites and BMI was assessed. The analysis was restricted to control samples to reduce collider bias, and one sample was randomly chosen from among duplicates. For each of the 117 metabolites, Pearson correlation coefficients were computed between BMI and, in turn, the imputed measurements, the normalized measurements, and the normalized measurements produced by a simpler normalization approach, which corrected for study and batch effects without attempting to preserve variation due to study center, BMI, gender, and alcohol intake. As displayed in Figure 6, most correlation values were above the line y = x, especially for values greater than 0.1: associations with BMI were stronger when using normalized data implementing our approach compared to those observed with both non-normalized data and normalized data implementing a simple, yet incomplete, normalization approach.

## 3. Discussion

In this work, a pipeline for the normalization of metabolomics data acquired in different studies was described. After a screening of informative metabolites and samples, the PC-PR2 method was used to identify major sources of variation in metabolomics data and linear mixed effect models were used to correct for unwanted sources of variation, while attempting to preserve biological variation and accounting for potential heteroscedasticity. The pipeline was applied to targeted metabolomics data acquired in eight cancer-specific case-control studies nested within EPIC. Substantial inter-study and inter-batch heterogeneity was observed in the original data. Accordingly, the technical reproducibility was low-to-moderate for many metabolites with ICC values lower than 0.50, especially for the semi-quantified metabolites (e.g., glycerophospholipids), suggesting that quantified metabolites might be less prone to unwanted variations due to analytical factors. Our normalization approach eliminated most of the inter-study and inter-batch variability and improved the technical reproducibility of a large proportion of semi-quantified and quantified metabolites, with most ICC values greater than 0.75. Normalization using the ComBat approach [19], which relies on a similar model but uses empirical Bayes estimation, performed similarly for all metabolites except acylcarnitines, for which ICC values were larger with our approach than ComBat. Normalization using the PCA-based method produced lower ICC values for most metabolites. All together, these results suggested that our approach outperformed ComBat and the PCA-based method on the EPIC metabolomics data. ICC values estimated from the duplicate samples originating from different blood matrices (serum versus citrate plasma) were generally larger than 0.75 after normalization. However, they were also generally lower than values estimated from all duplicate samples. In particular, the ICC for methionine was 0.39 (95% confidence interval, CI: 0.14–0.57), as compared to 0.71 (95% CI: 0.64–0.77) when ICC estimation used all duplicate samples. This result calls for caution when pooling samples originating from different blood matrices, as large differences were reported for specific metabolite concentrations in serum and plasma samples [28]. 

As samples within each individual EPIC study were all assayed in the same laboratory with the same LC-MS instruments, and mostly originated from the same blood matrix (except for GLBD that included serum and heparin plasma samples and BREA that included EDTA and citrate plasma samples), the variability due to these factors was encompassed into the inter-study variability and could not be assessed by the PC-PR2 analysis. In particular, although the large inter-study variability in the non-normalized data supported the presence of inter-laboratory and inter-instrument variability, as previously reported for the AbsoluteIDQ p180 kit [17], correction for batch and study effects also corrected for effects due to blood matrix and LC-MS instruments, which were both observed to contribute to less than 0.1% of the total variation in the normalized data. However, the inter-study and inter-batch variability also reflected biological variability, because factors like study center, gender, BMI, and alcohol intake were not equally distributed across studies and batches. Consequently, some of the biological variation due to these factors would be removed if the normalized data were simply computed as the residuals in linear mixed models adjusted for study and batch. Conversely, by accounting for study center, gender, BMI, and alcohol intake in the mixed models and by computing the normalized residuals using the step described in expression (2) in Section 4.2.4, the normalization preserved (some of) the variation due to these factors. This was illustrated by the distribution of normalized data that was shifted in opposite directions for studies including only men or women, and by the stronger associations with BMI observed when using the complete model for normalization compared to the simpler version that only included batch and study as random effects.

A critical step of normalization procedures that use linear mixed models, or more generally models with location/scale adjustments [19], is the choice of (i) factors that may generate unwanted variation, for which a correction should be implemented, and (ii) factors that represent biological variability, which should be preserved after normalization. As illustrated in Section 4.2.4, while the list of variables in (i) should be included in matrix X (like study and batch), variables in (ii) should be included in matrix Z, and the choice depends on the study design and on the ultimate objective of the analysis. If the objective is to identify metabolites associated with a given phenotype, e.g., BMI, it is crucial to include BMI in matrix Z, particularly if BMI is associated with specific variables included in matrix X. Conversely, if the ultimate objective of the study is to identify metabolites associated with, say, alcohol, while controlling for BMI, then alcohol should be included in matrix Z (particularly if it is associated with specific variables included in matrix X), but BMI could be included in matrix X, so that the associations are adjusted for BMI. In any case, performing sensitivity analyses with normalized data generated including different sets of variables in matrices X and Z is a good practice. 

In multicenter investigations like EPIC, study center is a sensitive variable as it expresses technical (preanalytical) variation, likely the result of specific procedures for blood collection, sample treatment, and storage, as well as biological variation reflecting specific lifestyle exposures, often characterized by geographical gradients. In addition, in a multicenter context, the relationship between two sets of variables could be evaluated at the overall level, at the center level, or at the individual level [29]. In this study, to use the whole variability in metabolomics and BMI data, center was initially included in matrix Z. In the sensitivity analysis, study center was included in matrix X and the center-specific variability was removed. As shown in Supplementary Figure S6, results were similar to the overall analysis suggesting that group-level correlations were similar to individual–level correlations [29]. Alternative methods, like SVA [20,28] and RRmix [29], use linear (mixed) models with latent variables to estimate variability attributed to unspecified sources of variation, ultimately to be removed. These methods do not require prior knowledge of the sources of unwanted variation, but require the identification of sources of biological variation, as their effects would likely be removed if not properly accounted for in the linear predictor of the model.

The decision to implement data normalization largely depends on the ultimate objectives of the analysis. As the relationship between metabolites and cancer risk is generally quantified in conditional logistic regression models for matched case-control studies, metabolite measurements are compared within each matched case-control pair. If cases and controls are assayed within the same batch (as was the case in the EPIC metabolomics data), the effects of study and batch on the means of the measurements are not a concern and normalization is not required unless the variances of the measurements also vary across studies or batches. However, if the evaluation focuses on the investigation of lifestyle determinants of metabolomics data, as for example in mediation analysis, the matching is “broken” and control for inter-batch and inter-study variability is required [7].

Although originally developed for the normalization of metabolomics data acquired in different studies, our pipeline could be used for data acquired in a single study, for example to correct for inter-batch variability while preserving biological variability and to correct for potential heteroscedastic structures of concentration levels across batches. Our pipeline could also be adapted to the normalization of biomarker data and other molecular data, possibly with some modifications. In particular, for the normalization of untargeted LC-MS metabolomics data, a step to exclude features based on comparison with blank samples should be added to the data cleaning [16], and a *K*-nearest neighbors approach has been shown to perform particularly well for the imputation of missing data [15,30] in the context of untargeted metabolomics data. Importantly, when processing untargeted metabolomics data from individual studies separately, different feature identifiers (e.g., mass to charge ratio and retention time) would characterize the same molecule in each study. Therefore, the pooling of several untargeted datasets would generally require an additional feature alignment step consisting of identifying the features present in the different datasets, which might be particularly challenging with data acquired in different laboratories [31].

With the increasing availability of metabolomics data in large scale epidemiological investigations, such as those participating in the COnsortium of METabolomics Studies (COMETS) [32], pooling will be more and more relevant as a strategy for increasing the statistical power when investigating the relationship between metabolomics data with disease indicators, environmental exposures, and/or other-omics and biomarker data. Combined with analytical and graphical inspection of the data to determine sources of unwanted variability to be removed and sources of biological variability to be preserved, linear mixed models provide a flexible tool to normalize metabolomics data, and possibly other -omics and biomarker data, prior to pooling data from different studies. As the comparability of measurements across studies is improved, our normalization approach could also be useful for studies that aim at the meta-analysis of individual patient data from different studies, in particular if heteroscedastic patterns of variability were observed.

## 4. Materials and Methods

### 4.1. The EPIC Study

EPIC is a large prospective study of over 500,000 men and women recruited in 1992–2000 in 23 centres in 10 European countries [23], originally designed to investigate the relationship between diet and cancer risk. Incident cancer cases were identified through a combination of methods including linkage to health insurance records, cancer, and pathology registries and active follow-up through study participants and their next-of-kin [23]. Around 386,000 participants from all countries provided a blood sample at recruitment. Fasting before blood withdrawal was not required. Blood was collected according to a standardized protocol in France, Germany, Greece, Italy, the Netherlands, Norway, Spain, and the UK [23]. Serum (except in Norway), plasma, erythrocytes, and buffy coat aliquots were stored in liquid nitrogen (−196 °C) in a centralized biobank at IARC. In Denmark, blood fractions were stored locally in the vapor phase of liquid nitrogen containers (−150 °C), and in Sweden, they were stored locally at −80 °C in standard freezers. Our analyses used targeted metabolomics data collected within the EPIC study and generated through the AbsoluteIDQ p180 or p150 commercial kit (Biocrates Life Science AG, Innsbruck Austria).

All participants provided written informed consent to participate in the EPIC study. This study was approved by the ethics committee of the International Agency for Research on Cancer (IARC) and all centers.

### 4.2. The Pipeline to Normalize Data

**Figure 7:**
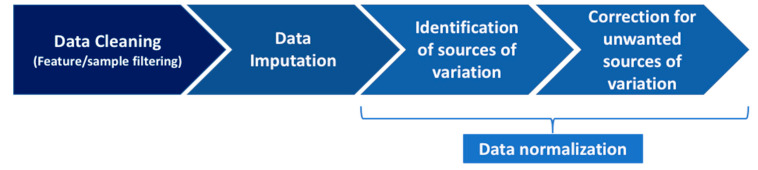
Main steps of the pipeline.

Given a matrix of *p* metabolites acquired on *n* samples, our pipeline implemented four main steps, as summarized in Figure 7 and detailed hereafter for the EPIC targeted metabolomics data. R scripts implementing these four steps, along with Rmarkdown and html documents that can be used to reproduce the analysis of the EPIC data, are available at https://code.iarc.fr/viallonv/pipeline_biocrates, accessed on 14 September 2021.

#### 4.2.1. Step 1: Data Cleaning

The objective of data cleaning was to remove the least informative metabolites and samples, using a number of (subjective) criteria. First, the pipeline excluded metabolites and samples exceeding a certain threshold of missingness (e.g., 20%) in each study separately. Missing values were here defined as fully missing values, for which no information on the real value was available. In particular, they did not include out of measurable range values, which corresponded to values that were missing because they were below the batch-specific limit of detection (LOD), below the kit-specific lower limit of quantification (LLOQ), or above the kit-specific upper limit of quantification (ULOQ). An extra step was implemented to exclude outlying samples within each batch based on principal component analysis (PCA) [11], using a 20% proportional expansion of the Hotellings T2 distribution ellipse, with the level of the ellipse set to 100 × (1 − 0.05)/N_b_% and N_b_ the total number of batches. Samples assayed in batches with less than 10 samples were also excluded to ensure enough information during batch-specific data imputation (Section 4.2.2) and normalization (Section 4.2.4).

#### 4.2.2. Step 2: Data Imputation

All missing values, including the out of measurable range values, were imputed in the cleaned dataset in each batch separately. Values below batch-specific LOD, below kit-specific LLOQ, or above kit-specific ULOQ were set to LOD/2, LLOQ/2, and ULOQ, respectively. Values below an unknown batch-specific LOD were set to LOD/2 after setting batch-specific LOD to study-specific medians of known LOD values. Fully missing values were set to the batch-specific median of non-missing values if less than 50% of the measurements in the batch were missing and to the study-specific median of the batch-specific medians otherwise. Measurements were log-transformed to reduce skewness.

#### 4.2.3. Step 3: Data Normalization, Part 1: Identification of Sources of Variation

The PC-PR2 technique was used to identify main sources of variation in the metabolomics data [13]. The PC-PR2 is a multivariate technique that combines PCA with multiple linear regression to assess the proportion of the variability of the full metabolomics dataset explained by a set of explanatory variables, including samples characteristics (age, sex, BMI, alcohol consumption, study center), as well as preanalytical and analytical factors (fasting status, sample processing protocol, blood matrix, study, batch, laboratory instrument). While the former set of factors likely determined biological variability, the latter set likely introduced sources of unwanted variation in metabolomics data. PCA was conducted on metabolite measurements and a number K ≥ 1 of components sufficient to explain more than 80% of total variability was retained. Component scores were, in turn, regressed on the list of aforementioned independent variables, say W_1_,…, W_Q_, in multiple linear regression models, and the partial R^2^ for each covariate W_q_ was estimated for each component (C_k_). For example, the partial-R^2^ for W_1_ conditional on the (Q-1) other covariates for component k was

R^2^_partial, k_ (W_1_) = [SSE(C_k_|W_2_,…, W_Q_) − SSE(C_k_|W_1_,W_2_,…, W_Q_)]/ SSE(C_k_|W_2_,…, W_Q_),

with SSE(C_k_|W_j_,…, W_Q_) expressing the residual sum of squares in the linear regression model of component C_k_ on variables W_j_,…, W_Q_, for j = 1, 2. For variables with a nested structure, for example study (S) and batch within study (B), the formula was

R^2^_partial, k_ (S) = [SSE(C_k_|W_2_,…, W_Q_) − SSE(C_k_|S, W_2_,…, W_q_)]/ SSE(C_k_|W_2_,…, W_Q_),

R^2^_partial, k_ (B) = [SSE(C_k_|S,W_2_,…, W_Q_) − SSE(C_k_|B, S,W_2_,…, W_Q_)]/ SSE(C_k_|S,W_2_,…, W_Q_).

An overall R^2^_partial_ (W_1_) was obtained by the average of terms R^2^_partial,k_ (W_1_) weighted by the eigenvalue of each component. This overall estimate provides a measure of the variability in the ensemble of metabolite concentrations that each explanatory variable contributes to explaining. The PC-PR2 technique is implemented in the pcpr2 R package available on GitHub.

#### 4.2.4. Step 4: Data Normalization, Part 2: Correction for the Unwanted Sources of Variation

In order to correct for unwanted sources of variability while preserving biological variability, a random effects model was used for each metabolite separately [14], as

*y* = *α* + *Xβ* + *Zθ* + *ε*, (1)

where *y* is the n-vector of the measurements for the metabolite/feature under consideration (all studies combined), the matrix *X* expresses variables corresponding to sources of variations that should be corrected for, and the optional matrix *Z* expresses variables corresponding to biological variations that should be preserved. Variables expressed in matrices *X* and *Z* typically include some of the variables W_1_, …, W_Q_ of the PC-PR2 analysis with largest R^2^_partial_. The vector of parameters *β* associated to matrix *X* may include both fixed- and random-effects, while the vector *θ* associated to matrix *Z* contains fixed effects only. Parameter *α* is the intercept and vector *ε~ N*_n_(*0,* *Σ*) corresponds to the random error of the model. Residuals *ε* are independent of the random effects of the model. Random effects are Gaussian, centered, and were further assumed to have diagonal covariance matrix in our illustration.

Parameters *α,* *β*, *θ*, and the vector of residuals *ε* under model (1) are estimated by, say, *a*, *b, c*, and *e*. Normalized residual measurements are computed as

*u* = *e* + *Zc*. (2)

In this way, normalization preserves the association between the metabolite and variables in *Z*, while any association with variables in *X* is eliminated. As mentioned in the Discussion, variables describing biological variations of interest should be included in matrix *Z* if they are associated with variables included in matrix *X* (e.g., sources of biological variations that are unequally balanced across studies or batches); otherwise, some of the variation they explain would be removed because of the adjustment for *X*. In our illustration, study center indicators, gender, body mass index, and alcohol intake were included in matrix *Z*, while batch and study indicators were included in matrix *X*.

In the simple homoscedastic random effect models, each component of the vector *ε* of residuals has the same variance: *Σ* = σ^2^*I_n_* for some σ^2^ > 0, where we denote by *I_p_* the identity matrix of size *p* for any positive integer *p*. However, in practice, pre-analytical and/or analytical factors may not only influence the means of the measurements via the term *Xβ* in model (1), but also their variance. For example, variances of components of *ε* may vary across studies. This was accounted for by working under heteroscedastic random effect models with a specified structure for the variance matrix *Σ* of the residuals, e.g., *Σ* was made of blocks of the form σ_s_^2^*I_ns_* for observations corresponding to study s (with *ns* the number of observations in study s). Then, residuals *e* were replaced by the Pearson residuals in Equation (2), after rescaling them to ensure that their overall variance equals that of the standard residuals. Homoscedastic models were implemented with the lmer function of the lme4 R package, while heteroscedastic models were implemented with the lme function of the nlme R package, using the *weights* instruction to specify the within-group heteroscedasticity structure.

For comparison, we also considered the ComBat method [19] of the sva R package [20], under which a fixed-effects version of model (1) is estimated using an empirical Bayes approach, to leverage the fact that sources of variation may affect many metabolites in similar ways. In our illustration, ComBat was applied to correct for batch effect (which also accounts for study effect), while attempting to preserve variations due to study center, gender, body mass index, and alcohol intake. We also considered a PCA-based method [21,22], where (i) a PCA was performed on the full metabolomics data matrix, and (ii), normalized measurements were the residuals obtained after regressing each metabolite on the first *K =* 2 principal components of the PCA; other choices for the value of parameter *K* were considered and led to similar or lower reproducibility (quantified by the intraclass correlation coefficient; see Section 4.3).

### 4.3. Computation of the Intraclass Correlation Coefficient Using Duplicated Samples

The EPIC data included duplicate samples corresponding to aliquots of a baseline blood sample from the same subject measured twice in different batches or in different studies. These duplicated samples were used to assess the technical reproducibility of metabolomics measurements, and in particular to compare technical reproducibility before and after normalization. In sensitivity analyses, ICC values were estimated using only duplicate samples originating from distinct blood matrices (serum and citrate plasma). For each metabolite, we estimated its ICC using a linear mixed effects model of the form [16]

*m_ik_* = *γ_i_* + *ξ_ik_* (3)

where *m_ik_* is the *k*-th replicate measurement of subject *i*, *k =* 1, 2, *γ_i_~N(μ,* *σ_γ_^2^)* is a subject-specific random effect (with *μ* corresponding to the general mean of *m_ik_*), *ξ_ik_~N(0,* *σ_ξ_^2^)* is the residual random error for replicate *k* of subject *i*, and Cov(*ξ_ik_*, *γ_i_*) = 0. Under this model, ICC = Var(*γ_I_*)/Var(*γ_I_ +* *ξ_ik_*), so the ICC estimate was defined as the ratio of the estimated between-subject variance to the estimated total variance (between- and within-subject). Model (3) above can be estimated even if only a portion of the subjects have replicated samples. It was implemented using the lmer function of the lme4 R package, and 95% confidence intervals (CI) of the ICC values were derived using the parametric bootstrap implemented by the bootMer function of the lme4 R package.

## Figures and Tables

**Table 1:** Main characteristics of the study population.

Acronym	Number of Samples	Matrix	Laboratory	MS Instrument	LC Instrument	Kit Used
BREA	3172	Citrate plasma ^1^	IARC	SCIEX QTRAP 5500	Agilent 1290	p180
CLRT1	946	Citrate plasma	IARC	SCIEX Triple Quad 4500	Agilent 1290	p180
CLRT2	2295	Serum	HZM ^3^	SCIEX API 4000	Agilent 1200	p150
ENDO	1706	Citrate plasma	ICL ^4^	SCIEX API 4000	Agilent 1290	p180
GLBD	112	Serum ^2^	HZM ^3^	SCIEX API 4000	Agilent 1200	p180
LIVE	662	Serum	IARC	SCIEX QTRAP 5500	Agilent 1290	p180
KIDN	1213	Citrate plasma	IARC	SCIEX QTRAP 5500	Agilent 1290	p180
PROS	6020	Citrate plasma	IARC	SCIEX Triple Quad 4500	Agilent 1290	p180

^1^ except Swedish participants (*n* = 101; EDTA plasma). ^2^ except for Swedish participants (*n* = 14, heparin plasma). ^3^ Helmhotz Zentrum München. ^4^ Imperial College London.

## Data Availability

The codes used in this analysis are available at https://code.iarc.fr/viallonv/pipeline_biocrates (accessed on 14 September 2021). EPIC data and biospecimens are available for investigators who seek to answer important questions on health and disease in the context of research projects that are consistent with the legal and ethical standard practices of IARC/WHO and the EPIC Centres. The primary responsibility for accessing the data belongs to IARC and the EPIC centres. Access to materials from the EPIC study can be requested by contacting epic@iarc.fr.

## Supplementary Materials

The following are available online at https://www.mdpi.com/article/10.3390/metabo11090631/s1, Figure S1: Correlations between normalized measurements produced by ComBat and our approach, Figure S2: Correlations between normalized measurements produced by the PCA-based method and our approach, Figure S3: Average ICC values for each class of metabolites after normalization using ComBat and our approach, Figure S4: Average ICC values for each class of metabolites after normalization using the PCA-based method and our approach, Figure S5: Metabolite-specific ICC values before and after normalization and average ICC values for each class of metabolites before and after normalization, Figure S6: Correlations (absolute values) between BMI and the 117 metabolites in control samples, Table S1: List of the 117 metabolites retained after the data cleaning step, Table S2: Study origin of duplicate samples in the EPIC targeted metabolomics data.
