# Supplementary material for: A New Pipeline for the Normalization and Pooling of Metabolomics Data
Source: Metabolites. 2021 Sep 17;11(9):631. doi: 10.3390/metabo11090631 (PMC8467830; doi:10.3390/metabo11090631)
Supplement: Supplementary file 1 [file metabolites-11-00631-s001.zip › metabolites-1321621-supplementary.pdf]

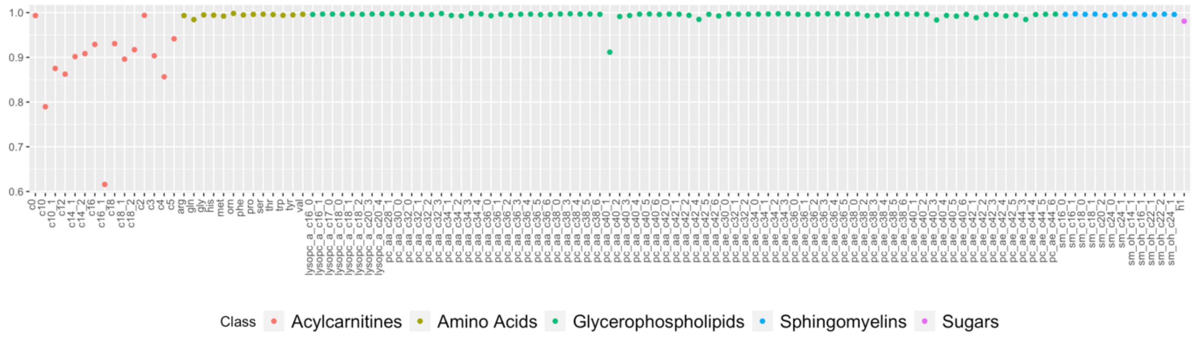

Supplementary Figure S1. Correlations between normalized measurements produced by ComBat and our approach. Both approaches were run to correct for batch and study effects, and to preserve biological variations due to study center, gender, alcohol intake and body mass index. The *y*-axis represents the correlation level, and the *x*-axis the 117 metabolites.

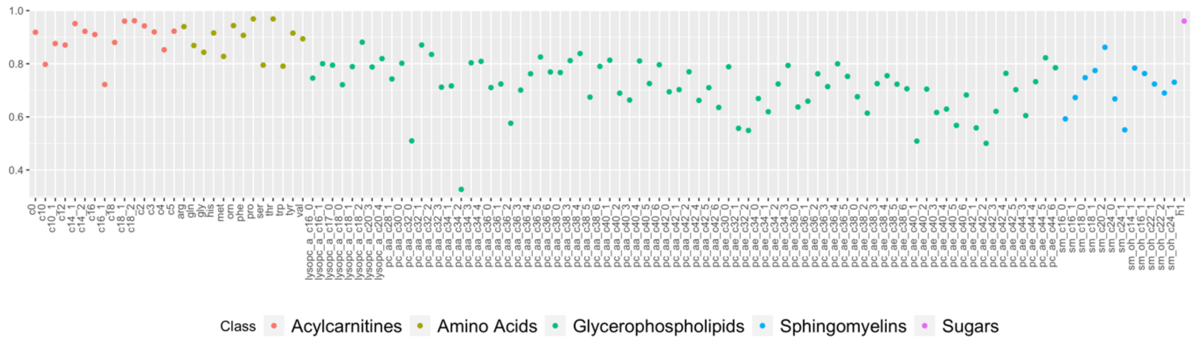

Supplementary Figure S2. Correlations between normalized measurements produced by the PCA-based method and our approach. Our approach was run to correct for batch and study effects, and to preserve biological variations due to study center, gender, alcohol intake and body mass index. The *y*-axis represents the correlation level, and the *x*-axis the 117 metabolites.

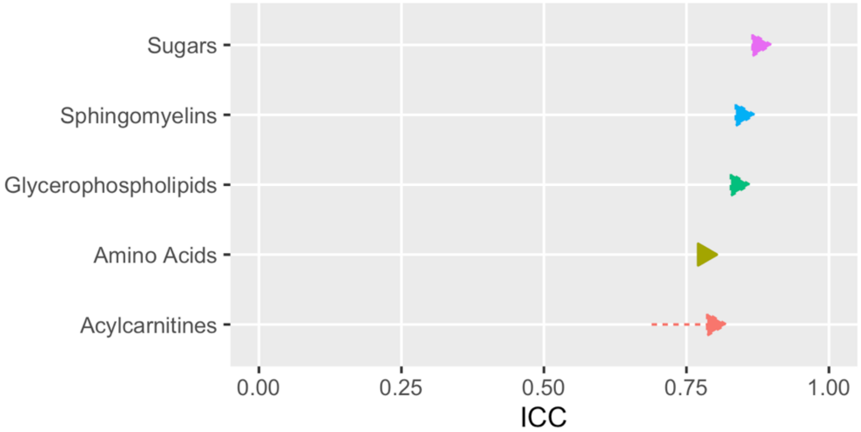

Supplementary Figure S3. Average ICC values (*x*-axis) for each class of metabolites (*y*-axis) after normalization using ComBat and our approach; for each arrow, its origin represents the ICC obtained when using ComBat and its peak represents the ICC obtained when using our approach. All arrows are oriented towards the right, especially for acylcarnitines, indicating that our approach produced more reproducible measurements for most metabolites.

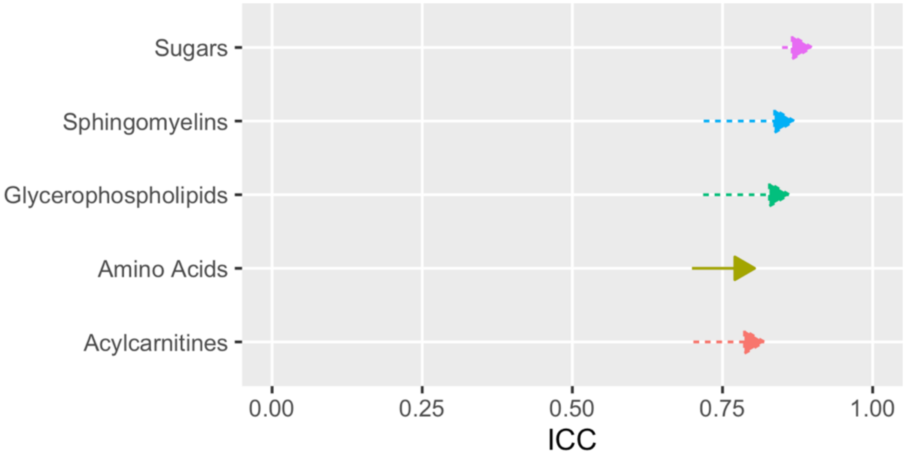

Supplementary Figure S4. Average ICC values (*x*-axis) for each class of metabolites (*y*-axis) after normalization using the PCA-based method and our approach; for each arrow, its origin represents the ICC obtained when using the PCA-based method and its peak represents the ICC obtained when using our approach. All arrows are oriented towards the right, indicating that our approach produced more reproducible measurements for most metabolites.

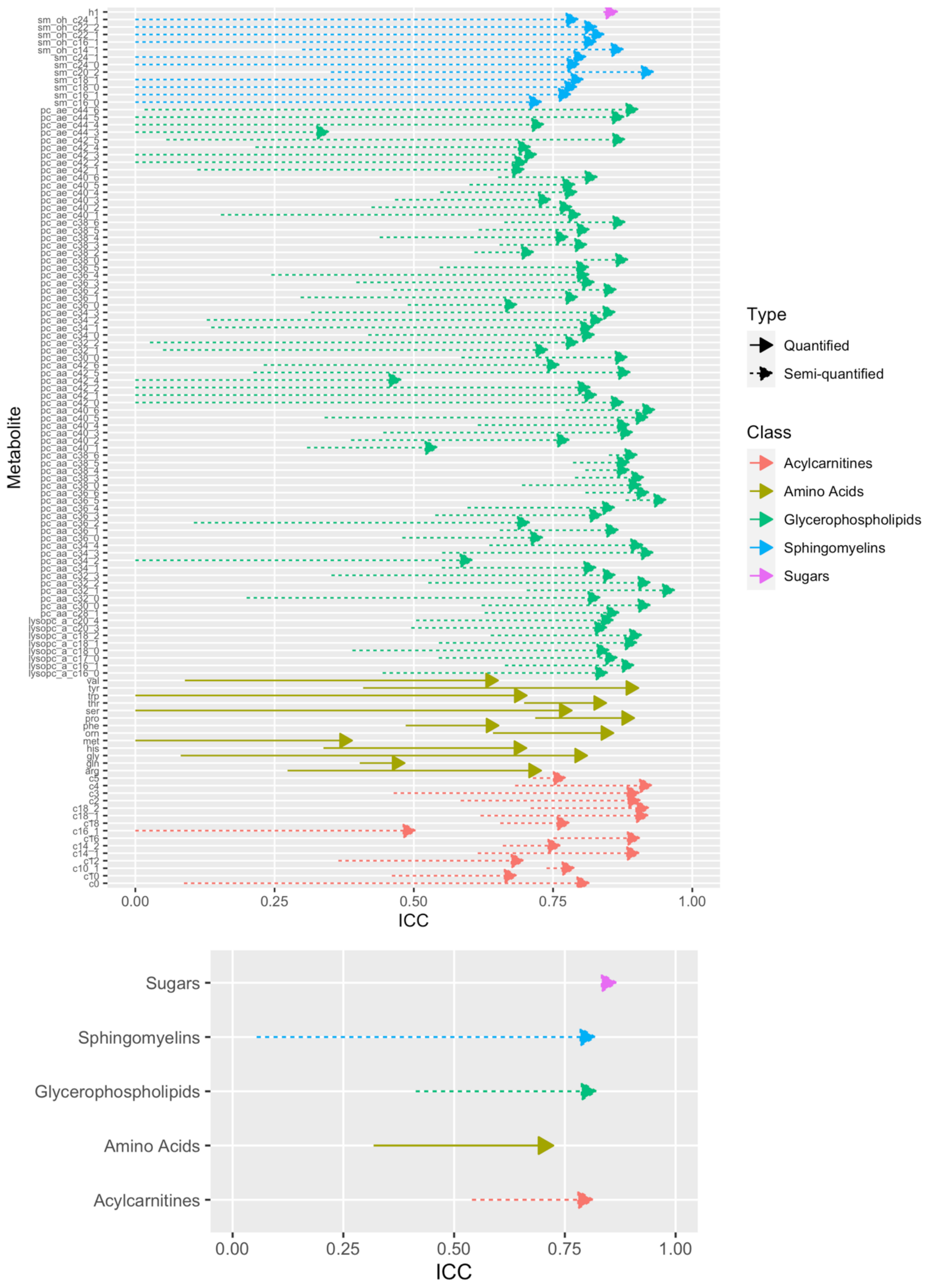

Supplementary Figure S5. Metabolite-specific ICC values before and after normalization (top) and average ICC values for each class of metabolites before and after normalization (bottom); normalization was conducted so as to remove study and batch effects while preserving variation due to study center, BMI, gender and alcohol intake. Only duplicate samples measured in two different studies and originating from two different blood matrices (serum and citrate plasma) were used here. For each arrow, its origin represents the ICC value before normalization, and its peak represents the ICC value after normalization. In each plot, the x-axis represents the ICC value, and the y-axis each particular metabolite (top) or class of metabolites (bottom).

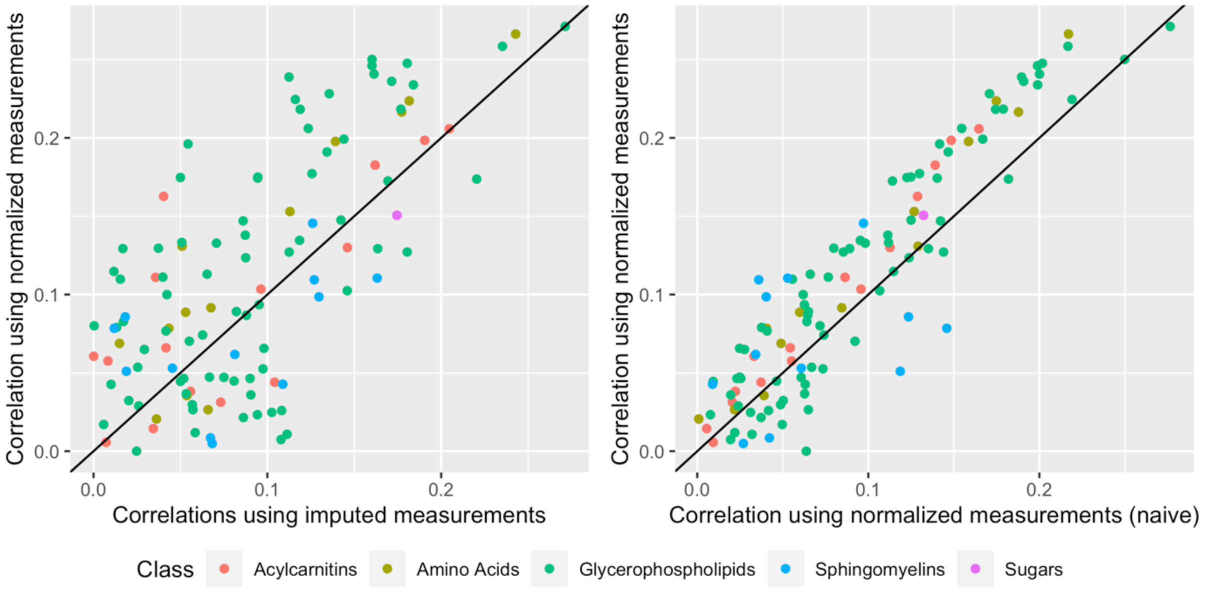

Supplementary Figure S6. Correlations (absolute values) between BMI and the 117 metabolites in control samples. The *y*-axis represents values computed with normalized measurements (the normalization was run so as to remove study, batch and center effects while preserving variation due to BMI, gender and alcohol intake), while the *x*-axis represents values computed with imputed (non-normalized) measurements (left), and normalized measurements produced by the "naïve" normalization (right), which corrects for study, batch and center effects without preserving variation due to BMI, gender and alcohol intake.

Supplementary Table S1. List of the 117 metabolites retained after the data cleaning step.

Name	Symbol in Figures	Class
Carnitine	c0	Acylcarnitins
Acetylcarnitine	c2	Acylcarnitins
Propionylcarnitine	c3	Acylcarnitins
Butyrylcarnitine	c4	Acylcarnitins
Valerylcarnitine	c5	Acylcarnitins
Decanoylcarnitine	c10	Acylcarnitins
Decenoylcarnitine	c10_1	Acylcarnitins
Dodecanoylcarnitine	c12	Acylcarnitins
Tretradecenoylcarnitine	c14_1	Acylcarnitins
Tetradecadienlycarnitine	c14_2	Acylcarnitins
Hexadecanoylcarnitine	c16	Acylcarnitins
Hexadecenoylcarnitine	c16_1	Acylcarnitins
Octadecanoylcarnitine	c18	Acylcarnitins
Octadecenoylcarnitine	c18_1	Acylcarnitins
Octadecadienylcarnitine	c18_2	Acylcarnitins
Arginine	arg	Amino Acids
Glutamine	gln	Amino Acids
Glycine	gly	Amino Acids
Histidine	his	Amino Acids
Methionine	met	Amino Acids
Ornithine	orn	Amino Acids
Phenylalanine	phe	Amino Acids
Proline	pro	Amino Acids
Serine	ser	Amino Acids
Threonine	thr	Amino Acids
Tryptophan	trp	Amino Acids
Tyrosine	tyr	Amino Acids
Valine	val	Amino Acids
lysoPC a C16:0	lysopc_a_c16_0	Glycerophospholipids
lysoPC a C16:1	lysopc_a_c16_1	Glycerophospholipids
lysoPC a C17:0	lysopc_a_c17_0	Glycerophospholipids
lysoPC a C18:0	lysopc_a_c18_0	Glycerophospholipids
lysoPC a C18:1	lysopc_a_c18_1	Glycerophospholipids
lysoPC a C18:2	lysopc_a_c18_2	Glycerophospholipids
lysoPC a C20:3	lysopc_a_c20_3	Glycerophospholipids
lysoPC a C20:4	lysopc_a_c20_4	Glycerophospholipids
PC aa C28:1	pc_aa_c28_1	Glycerophospholipids
PC aa C30:0	pc_aa_c30_0	Glycerophospholipids
PC aa C32:0	pc_aa_c32_0	Glycerophospholipids

Supplementary Table S1 (continued)

Name	Symbol in Figures	Class
PC aa C32:1	pc_aa_c32_1	Glycerophospholipids
PC aa C32:3	pc_aa_c32_3	Glycerophospholipids
PC aa C34:1	pc_aa_c34_1	Glycerophospholipids
PC aa C34:2	pc_aa_c34_2	Glycerophospholipids
PC aa C34:3	pc_aa_c34_3	Glycerophospholipids
PC aa C34:4	pc_aa_c34_4	Glycerophospholipids
PC aa C36:0	pc_aa_c36_0	Glycerophospholipids
PC aa C36:1	pc_aa_c36_1	Glycerophospholipids
PC aa C36:2	pc_aa_c36_2	Glycerophospholipids
PC aa C36:3	pc_aa_c36_3	Glycerophospholipids
PC aa C36:4	pc_aa_c36_4	Glycerophospholipids
PC aa C36:5	pc_aa_c36_5	Glycerophospholipids
PC aa C36:6	pc_aa_c36_6	Glycerophospholipids
PC aa C38:0	pc_aa_c38_0	Glycerophospholipids
PC aa C38:3	pc_aa_c38_3	Glycerophospholipids
PC aa C38:4	pc_aa_c38_4	Glycerophospholipids
PC aa C38:5	pc_aa_c38_5	Glycerophospholipids
PC aa C38:6	pc_aa_c38_6	Glycerophospholipids
PC aa C40:1	pc_aa_c40_1	Glycerophospholipids
PC aa C40:2	pc_aa_c40_2	Glycerophospholipids
PC aa C40:3	pc_aa_c40_3	Glycerophospholipids
PC aa C40:4	pc_aa_c40_4	Glycerophospholipids
PC aa C40:5	pc_aa_c40_5	Glycerophospholipids
PC aa C40:6	pc_aa_c40_6	Glycerophospholipids
PC aa c42:0	pc_aa_c42_0	Glycerophospholipids
PC aa c42:1	pc_aa_c42_1	Glycerophospholipids
PC aa C42:2	pc_aa_c42_2	Glycerophospholipids
PC aa C42:4	pc_aa_c42_4	Glycerophospholipids
PC aa C42:5	pc_aa_c42_5	Glycerophospholipids
PC aa C42:6	pc_aa_c42_6	Glycerophospholipids
PC ae C30:0	pc_ae_c30_0	Glycerophospholipids
PC ae C30:1	pc_ae_c32_1	Glycerophospholipids
PC ae C30:2	pc_ae_c32_2	Glycerophospholipids
PC ae C34:0	pc_ae_c34_0	Glycerophospholipids
PC ae C34:1	pc_ae_c34_1	Glycerophospholipids
PC ae C34:2	pc_ae_c34_2	Glycerophospholipids
PC ae C34:3	pc_ae_c34_3	Glycerophospholipids
PC ae C36:0	pc_ae_c36_0	Glycerophospholipids

Supplementary Table S1 (continued)

Name	Symbol in Figures	Class
PC ae C36:1	pc_ae_c36_1	Glycerophospholipids
PC ae C36:2	pc_ae_c36_2	Glycerophospholipids
PC ae C36:3	pc_ae_c36_3	Glycerophospholipids
PC ae C36:4	pc_ae_c36_4	Glycerophospholipids
PC ae C36:5	pc_ae_c36_5	Glycerophospholipids
PC ae C38:0	pc_ae_c38_0	Glycerophospholipids
PC ae C38:2	pc_ae_c38_2	Glycerophospholipids
PC ae C38:3	pc_ae_c38_3	Glycerophospholipids
PC ae C38:4	pc_ae_c38_4	Glycerophospholipids
PC ae C38:5	pc_ae_c38_5	Glycerophospholipids
PC ae C38:6	pc_ae_c38_6	Glycerophospholipids
PC ae C40:1	pc_ae_c40_1	Glycerophospholipids
PC ae C40:2	pc_ae_c40_2	Glycerophospholipids
PC ae C40:3	pc_ae_c40_3	Glycerophospholipids
PC ae C40:4	pc_ae_c40_4	Glycerophospholipids
PC ae C40:5	pc_ae_c40_5	Glycerophospholipids
PC ae C40:6	pc_ae_c40_6	Glycerophospholipids
PC ae C42:1	pc_ae_c42_1	Glycerophospholipids
PC ae C42:2	pc_ae_c42_2	Glycerophospholipids
PC ae C42:3	pc_ae_c42_3	Glycerophospholipids
PC ae C42:4	pc_ae_c42_4	Glycerophospholipids
PC ae C42:5	pc_ae_c42_5	Glycerophospholipids
PC ae C44:3	pc_ae_c44_3	Glycerophospholipids
PC ae C44:4	pc_ae_c44_4	Glycerophospholipids
PC ae C44:5	pc_ae_c44_5	Glycerophospholipids
PC ae C44:6	pc_ae_c44_6	Glycerophospholipids
SM C16:0	sm_c16_0	Sphingomyelins
SM C16:1	sm_c16_1	Sphingomyelins
SM C18:0	sm_c18_0	Sphingomyelins
SM C18:1	sm_c18_1	Sphingomyelins
SM C20:2	sm_c20_2	Sphingomyelins
SM C24:0	sm_c24_0	Sphingomyelins
SM C24:1	sm_c24_1	Sphingomyelins
SM (OH) C14:1	sm_oh_c14_1	Sphingomyelins
SM (OH) C16:1	sm_oh_c16_1	Sphingomyelins
SM (OH) C22:1	sm_oh_c22_1	Sphingomyelins
SM (OH) C22:2	sm_oh_c22_2	Sphingomyelins
SM (OH)C24:1	sm_oh_c24_1	Sphingomyelins
Hexoses	h1	Monosaccharides

Supplementary Table S2: Study origin of duplicate samples in the EPIC targeted metabolomics data.

Study 1	Study 2	Number of EPIC participants
BREA	CLRT1	1
BREA	CLRT2	4
BREA	ENDO	5
BREA	GLBD	1
BREA	LIVE	2
CLRT1	CLRT2	2
CLRT1	ENDO	2
CLRT1	KIDN	2
CLRT1	LIVE	1
CLRT1	PROS	4
CLRT2	ENDO	4
CLRT2	KIDN	5
CLRT2	PROS	27
ENDO	KIDN	2
GLBD	LIVE	51
GLBD	PROS	1
KIDN	LIVE	1
KIDN	PROS	23
LIVE	PROS	9
